# Health-related quality of life in infertile couples receiving IVF or ICSI treatment

**DOI:** 10.1186/1472-6963-8-186

**Published:** 2008-09-19

**Authors:** Batool Rashidi, Ali Montazeri, Fatemeh Ramezanzadeh, Mamak Shariat, Nasrin Abedinia, Mahnaz Ashrafi

**Affiliations:** 1 Vali-e-Asr Reproductive Health Research Center, Tehran University of Medical Sciences, Tehran, Iran; 2 Iranian Institute for Health Sciences Research, Tehran, Iran; 3 Public Health and Health Policy, Division of Community-Based Sciences, University of Glasgow, Glasgow, Scotland, UK; 4 Royan Institute, Tehran, Iran

## Abstract

**Background:**

Infertile couples might experience psychological distress and suffer from impaired health-related quality of life. This study aimed to examine health-related quality of life in infertile couples receiving either in-vitro fertilization (IVF) or intracytoplasmic sperm injection (ICSI) treatment.

**Methods:**

This was a cross-sectional study of quality of life in infertile couples attending to Vali-e-Asr Reproductive Health Research Center or Royan Institute for either IVF or ICSI treatment in Tehran, Iran. Health-related quality of life was assessed using the Short Form Health Survey (SF-36). Patients' demographic and clinical characteristics were also recorded. Data were analyzed to compare quality of life in infertile women and men and to indicate what variables predict quality of life in infertile couples.

**Results:**

In all 514 women and 514 men (n = 1028) were studied. There were significant differences between women and men indicating that male patients had a better health-related quality of life. Also health-related quality of life was found to be better in infertility due to male factor. Performing logistic regression analysis it was found that female gender, and lower educational level were significant predictors of poorer physical health-related quality of life. For mental health-related quality of life in addition to female gender and lower educational level, younger age also was found to be a significant predictor of poorer condition. No significant results were observed for infertility duration or causes of infertility either for physical or mental health-related quality of life.

**Conclusion:**

The findings suggest that infertility duration or causes of infertility do not have significant effects on health-related quality of life in infertile couples. However, infertile couples, especially less educated younger women, are at risk of a sub-optimal health-related quality of life and they should be provided help and support in order to improve their health-related quality of life.

## Introduction

Infertility is a major life crisis [1]. Infertility can cause depression, anxiety, social isolation and sexual dysfunction [2,3]. Due to this frustrating experience many infertile couples would seek medical help and finally will receive assisted reproductive treatment [4]. According to patients' conditions most patients receive in-vitro fertilization (IVF) or intracytoplasmic sperm injection (ICSI) treatment. Most couples that plan to have treatment experience extensive and emotionally challenging methods of diagnosis and treatment. Often, their treatment options are limited and the procedures are considered by many couples to be the end of the line. People who seek treatment for infertility have been reported to be more anxious and emotionally distressed than people in the general population [5]. It is still not clear whether this elevated level of distress occurs in all couples planning to undergo infertility treatment or certain sub-groups may have more problems. It is likely, for instance, that infertility has a different impact on young people than on relatively older people planning to undergo treatment since for such couples treatment might be their last chance of having a baby [6]. In addition, factors that predicting quality of life may vary in different infertile populations, different gender and different ethnic backgrounds. Thus, the identification of factors associated with better or worse health-related quality of life is vital in order to propose and test scientifically based interventions for infertile populations [7].

Despite the existence of an extensive body of the literature on psychological aspects of the infertility, there are quite a few studies on health-related quality of life in infertile couples. A review of literature indicated that since 1982 to 2008 there were about ten studies that focused on health-related quality of life in infertile couples. [8]. This study aimed to contribute to existing knowledge and shad more light on the topic by reporting from a developing country where infertility might cause several social problems for infertile couples especially for women [9].

## Methods

### Design and data collection

From March 2006 to July 2006 all infertile couples attending to Vali-e-Asr Reproductive Health Research Center or Royan Institute for IVF or ICSI treatment were asked to participate in this prospective, cross sectional survey. A total of 1028 patients (514 women, 514 men) were entered into the study. Couples completed the questionnaire separately in the hospital setting at the time of their first clinical visits at the same point in the process of treatment.

### Measures

Health-related quality of life was measured using the Short Form Health Survey-SF-36 [10]. The SF-36 includes eight subscales, and scores on each subscale range from zero to 100 indicating worse to better conditions respectively. The eight subscales are: Physical functioning (10 questions), Role physical (4 questions), Role emotional (3 questions) Pain (2 questions), Vitality (4 questions) General health (5 questions), Social functioning (2 questions), and Mental health (5 questions). The SF-36 provides two summary scores: physical component summary (PCS) and mental component summary (MCS). The psychometric properties of the instrument are well documented [11].

In addition, demographic and clinical characteristics of patients including age, gender, education, duration of infertility, previous treatment for infertility and infertility causes were also collected.

### Analysis

Descriptive analyses were carried out to explore the data. The distributions of the health-related quality of life scores were examined. The scores were slightly skewed but all skewness values (except for physical functioning) were less than one allowing for using parametric tests. Statistical procedures included chi-square test for categorical data and t-test and one-way analyses of variance (ANOVA) for continuous data to compare the differences between the study sample subgroups. Logistic regression analysis was preformed to examine the predictive value of independent variables studied. For the purpose of analysis we used the physical component summary (PCM) and mental component summary (MCS) scores and relative to the mean score, patients were divided into two groups those who scored equal or higher than mean and those who scores lower than mean. The analysis was adjusted for the variables studied.

### Ethics

The ethics committee of Tehran University of Medical Sciences approved the study. All patients gave consent for the study.

## Results

In all, 1028 male and female were entered into the study. The mean age of woman was 31.4 years (SD = 5.9) and for men it was 35.9 years (SD = 6.0). Table 1 gives an overview of the main characteristics of the study sample.

**Table 1:** The characteristics of the study sample

	Male (n = 514)	Female (n = 514)	P (χ^2^, df)
	No. (%)	No. (%)	

Age groups (years)			< 0.001 (117.2, 3)
< 25	3 (0.6)	65 (12. 6)	
25–30	98 (19.0)	180 (35.0)	
31–35	156 (30.4)	128 (25.0)	
> 35	257 (50.0)	141 (27.4)	
Mean (SD)	35.9 (6.0)	31. 4 (5.9)	
Educational status (years)			0.21 (3.09, 2)
≤ 5 (primary)	142 (27.6)	157 (30.5)	
6–12 (secondary)	193 (37.5)	204 (39.7)	
> 12 (higher)	179 (34.8)	153 (29.8)	
Duration of infertility (years)			0.99 (0.017, 2)
≤ 5	177 (34.4)	179 (34.8)	
6–10	163 (31.7)	162 (31.5)	
> 10	174 (33.9)	173 (33.7)	
Mean (SD)	8.8 (5.4)	8.8 (5.4)	
Previous treatment for infertility			< 0.0001 (14.7, 1)
No	343 (67)	283 (55)	
Yes	171 (33)	231 (45)	
Causes of infertility			0.85 (0.78, 3)
Female factor	111 (21.6)	122 (23.7)	
Male factor	292 (56.8)	286 (55.6)	
Both (male & female factor)	43 (8.4)	39 (7.6)	
Unexplained	68 (13.2)	67 (13.0)	

The SF-36 scores for females and males are shown in Table 2. Comparing males and females it was found that male significantly scored higher, indicating to have a better health-related quality of life.

**Table 2:** The SF-36 scores by gender

	Male (n = 514)	Female (n = 514)	P*	Normative data for males**	Normative data for females**
	Mean (SD)	Mean (SD)		Mean (SD)	Mean (SD)

PF	86.7 (20.9)	80.6 (21.8)	< 0.0001	87.8 (19.0)	82.9 (22.1)
RP	78.3 (31.0)	72.0 (33.3)	0.002	73.8 (36.4)	66.5 (39.1)
BP	77.4 (16.8)	69.1 (20.2)	< 0.0001	82.7 (23.4)	76.4(26.2)
GH	71.0 (17.1)	66.1 (18.1)	< 0.0001	70.2 (19.6)	65.0 (20.8)
SF	76.4 (21.9)	72.6 (22.8)	0.007	78.0 (23.5)	74.2 (25.1)
RE	73.0 (35.6)	65.4 (38.6)	0.001	70.1 (39.7)	61.4 (42.4)
VT	66.2 (17.6)	59.2 (18.5)	< 0.0001	68.9 (16.2)	62.9 (17.8)
MH	67.2 (17.8)	61.6 (19.6)	< 0.0001	69.2 (17.1)	65.0 (18.6)

* Derived from two independent samples t-test.

** Data for the general population aged 15 and above [11].

PF: physical functioning, RP: role physical, BP: bodily pain, GH: general health, SF: social functioning, RE: role emotional, VT: vitality, MH: mental health.

The health-related quality of life scores by infertility causes are shown in Table 3. There were significant differences on 3 measures (physical functioning, social functioning and mental health).

**Table 3:** The SF-36 scores by infertility causes (514 women and 514 men, higher scores indicate a better condition)

	Female factor (n = 233)	Male factor (n = 578)	Both (n = 82)	Unexplained (n = 135)	P*
	Mean (SD)	Mean (SD)	Mean (SD)	Mean (SD)	

PF	80.5 (23.8)	85.0 (20.5)	86.6 (17.1)	81.3 (23.5)	0.01
RP	71.4 (33.8)	77.0 (31.2)	78.3 (31.3)	71.8 (34.6)	0.06
BP	72.6 (18.9)	72.9 (19.6)	73.2 (18.4)	76.0 (17.4)	0.36
GH	66.1 (17.9)	69.5 (17.5)	68.3 (20.9)	69.0 (16.3)	0.09
SF	69.4 (23.8)	76.4 (21.6)	77.2 (22.7)	73.8 (22.3)	0.001
RE	63.9 (39.3)	70.8 (36.6)	73.5 (35.4)	69.3 (37.3)	0.07
VT	60.5 (19.2)	64.1 (17.8)	61.7 (18.7)	61.4 (18.7)	0.05
MH	60.7 (20.7)	65.8 (18.1)	66.2 (20.2)	63.9 (17.7)	0.005

* Derived from one-way analysis of variance (ANOVA)

PF: physical functioning, RP: role physical, BP: bodily pain, GH: general health, SF: social functioning, RE: role emotional, VT: vitality, MH: mental health.

Finally, to indicate independent effects of variables studied, logistic regression analysis was preformed. Table 4 indicates the results. Overall, younger age, female gender and lower level of education were found to be significant predictors of poorer health-related quality of life whereas duration of infertility, previous treatment for infertility or causes of infertility were not.

**Table 4:** Results of logistic regression analysis for poorer physical and mental health-related quality of life

	Adjusted OR (95% CI)	P
Physical component summary		
Age groups (years)		
> 35	1.0 (ref.)	
31–35	0.91 (0.62–1.32)	0.62
25–30	1.15 (0.82–1.61)	0.39
< 25	1.24 (0.67–2.26)	0.48
Sex		
Male	1.0 (ref.)	
Female	2.03 (1.53–2.68)	< 0.0001
Education		
> 12 (higher)	1.0 (ref.)	
6–12 (secondary)	1.40 (1.02–1.93)	0.03
≤ 5 (primary)	1.77 (1.25–2.51)	0.001
Infertility duration (years)		
> 10	1.0 (ref.)	
6–10	0.93 (0.65–1.33)	0.71
≤ 5	1.16 (0.84–1.61)	0.34
Previous treatment for infertility		
No	1.0 (ref.)	
Yes	1.06 (0.81–1.39)	0.67
Causes of infertility		
Unexplained	1.0 (ref.)	
Female factor	1.16 (0.74–1.80)	0.51
Male factor	0.76 (0.51–1.13)	0.18
Both	0.65 (0.36–1.18)	0.16
Mental component summary		
Age groups (years)		
> 35	1.0 (ref.)	
31–35	1.22 (0.85–1.76)	0.28
25–30	1.46 (1.05–2.03)	0.02
< 25	2.15 (1.16–3.97)	0.01
Sex		
Male	1.0 (ref.)	
Female	1.4 (1.07–1.85)	0.01
Education		
> 12 (higher)	1.0 (ref.)	
6–12 (secondary)	1.68 (1.19–2.36)	0.003
≤ 5 (primary)	1.35 (0.98–1.84)	0.06
Infertility duration (years)		
> 10	1.0 (ref.)	
6–10	0.99 (0.72–1.36)	0.94
≤ 5	1.10 (0.77–1.57)	0.60
Previous treatment for infertility		
No	1.0 (ref.)	
Yes	1.22 (0.93–1.58)	0.15
Causes of infertility		
Unexplained	1.0 (ref.)	
Female factor	1.38 (0.89–2.14)	0.14
Male factor	0.87 (0.59–1.29)	0.51
Both	0.70 (0.39–1.24)	0.22

## Discussion

This study investigated health-related quality of life of a large group of men and women planning to undergo IVF or ICSI treatment using the SF-36 questionnaire. The SF-36 scores were markedly lower in females as compared to males. The study reported by Ragni et al. also showed similar results for infertile Italian couples where men scored higher than woman on the SF-36 [12]. Several studies have suggested that the impact of infertility and its treatment is higher in women than men and also clearly demonstrated that having children was more important to women than men [6,13-17]. One reason for such findings is due to the fact that in general women usually rate their health-related quality of life lower than male gender. Another explanation is that women are blamed (or some times they take the blame) more frequently for the couple's infertility and thus, the stigma associated with such blaming (regardless of the diagnosis) causes more distress and deteriorations in health-related quality of life in female partners [9,18]. However, it should be noted that beyond general measures of health-related quality of life such as the SF, the scope of infertility and its effect on men might be different. For example, in a very recent study assessing sexual function and health-related quality of life in the male partner of infertile couples, Shindel et al. [19] reported that depression, erectile dysfunction and sexual relationship problems were prevalent among male partners of infertile couples and male partners reported significantly lower standardized scores on mental health compared to normative data. As suggested the evaluation of infertile women and men without comparing them to the general population contain several biases. Therefore it is recommended that when studying health-related quality of life in infertile couples it is better to include a control group to have a better understanding on the topic [12]. Unfortunately we were not able to provide such information and this requires a further investigation.

A study reported by Fekkes et al. showed that women planning IVF, in particular young women aged between 21–30 years, experienced more social and emotional problems than women of the same age group in the general population [6]. The present study also showed that younger age was a significant predictor for poorer mental health-related quality of life but not for physical health-related quality of life (see Table 4). It is argued that older patients have a longer history of infertility and thus might be able to cope with their medical situations in a better way because they have had more infertility-related experiences. However, it is argued that when the childlessness has become definite, psychological intervention should be continued to teach couples how to cope actively with their problems and how to ask for support in order to decrease the negative impact of their childlessness [20].

The study findings showed that lower educational level was a significant predictor of the poorer health-related quality of life, both physical and mental health. It is argued that certain socio-demographic characteristics of infertile women could be of importance in terms of perceived poorer health-related quality of life. For instance, it has been shown that highly educated infertile women would feel less stigmatized as compared to those with less education [9].

We also investigated the relationship between duration of infertility, causes of infertility and health-related quality of life. In univariate analysis we found that health-related quality of life was better in couples with male factor infertility or both male and female infertility factor (Table 3). However, the multivariate analysis showed that duration of infertility or causes of infertility were not significant predictors of poorer health-related quality of life (Table 4). As suggested these findings indicate that infertility might lead to similar experiences by all men and women, although they might explain themselves in different ways.

## Conclusion

The study findings suggest that duration of infertility or causes of infertility do not have significant effects on health-related quality of life in infertile couples. However, infertile couples, especially less educated younger women, are at risk of a sub-optimal health-related quality of life and they should be provided help and support in order to improve their health-related quality of life.

## Competing interests

The authors declare that they have no competing interests.

## Authors' contributions

BR was the main investigator and wrote the first draft. AM analyzed the data and wrote the final manuscript. FR, MS, and MA contributed to the study design, patient recruitments and helped to collect data. NA contributed to the data collection, data entry and coordinated the study. All authors read and approved the final manuscript.

## Pre-publication history

The pre-publication history for this paper can be accessed here:


